# Splenogonadal fusion - a rare cause of scrotal swelling: a case report

**DOI:** 10.1186/s13256-018-1712-1

**Published:** 2018-06-20

**Authors:** O. Karray, A. Oueslati, M. Chakroun, H. Ayed, A. Bouzouita, M. Cherif, M. R. Ben Slama, A. Derouiche, M. Chebil

**Affiliations:** Urology department, Charles Nicolle hospital, Tunis, Tunisia

**Keywords:** Testis, Congenital abnormalities, Spleen, Orchiectomy

## Abstract

**Background:**

Splenogonadal fusion is a rare and benign condition. Diagnosis is challenging for clinicians. Despite its indolence, diagnosis is often confirmed after orchidectomy. Surgery is mandatory, particularly to rule out the extremely rare association with malignancy.

**Case presentation:**

We report a case of splenogonadal fusion in a 38-year-old North African man presenting a palpable scrotal mass. We describe clinical aspects, pathogenic hypothesis, radiological features, as well as surgical management principles.

**Conclusions:**

Splenogonadal fusion is rarely suspected and diagnosed preoperatively. A diagnosis is made once an ectopic testicular mass is associated with cryptorchidism and suggestive radiological signs. A better knowledge of the clinical and radiological features of splenogonadal fusion provides an opportunity for conservative surgery.

## Background

Splenogonadal fusion is a rare congenital defect, defined by the presence of ectopic splenic tissue in the scrotum. It is usually discovered in adolescents with a scrotal swelling. As imaging findings are not distinctive, histological diagnosis is often made after radical orchidectomy. We report a new case of splenogonadal fusion in an adult patient presenting a palpable scrotal mass. Pathogenic and clinical features, imaging findings, and surgical management will be described and discussed.

## Case presentation

**Fig. 1:**
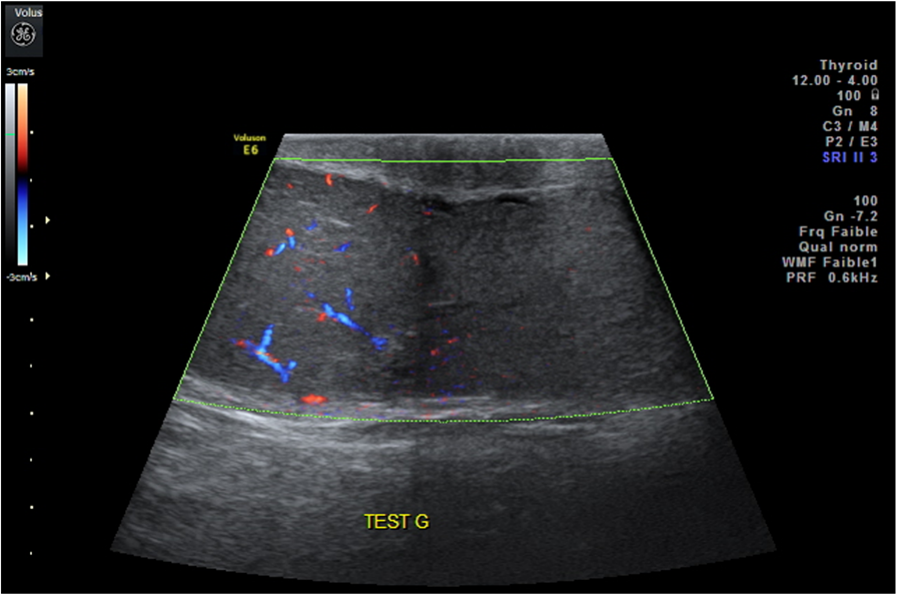
Scrotal ultrasound: poorly vascularized mass appended to the upper pole of the left testis

A 38-year-old North African man, with no past medical history, consulted our out-patient clinic for a painless left scrotal mass. There was no history of previous orchitis or scrotal contusion. He noted the mass a month ago. A physical examination found a 2 cm palpable mass in the upper pole of his left testis. There were no signs of scrotal inflammation. The mass had a firm consistency and regular margins. Palpation of his right testis and the lower pole of his left testis were normal. Routine blood tests were normal. As a testicular tumor was strongly suspected, a bioassay of testicular tumor markers was ordered. Alpha-fetoprotein, human chorionic gonadotropin (hCG), and lactate dehydrogenase (LDH) were in the normal ranges. There was no bacterial growth in urine analysis, including *Mycobacterium tuberculosis* screening. A scrotal ultrasound showed a homogeneous testicular parenchyma, with a conserved vascularization on Doppler. An extratesticular mass was observed, attached to the upper pole of his testis. The mass was isoechoic to the testis parenchyma, and poorly vascularized Doppler (Fig. 1).

**Fig. 2:**
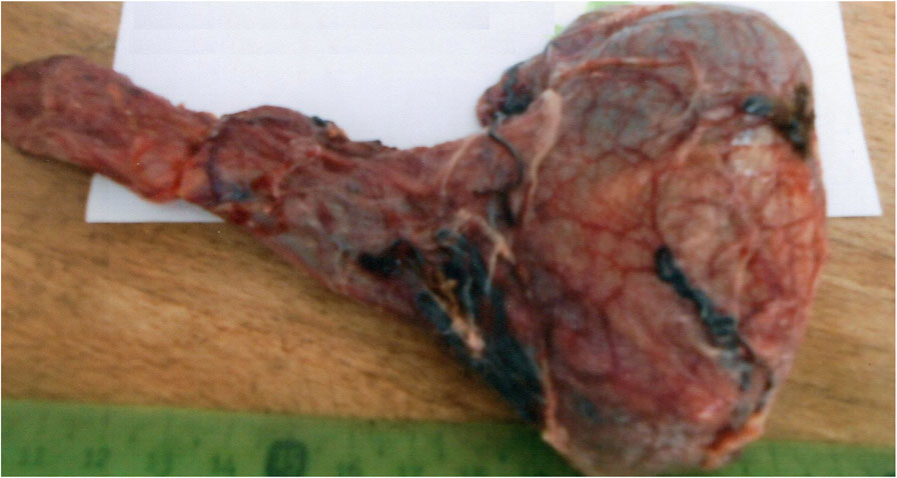
Operative specimen: left inguinal orchiectomy

**Fig. 3:**
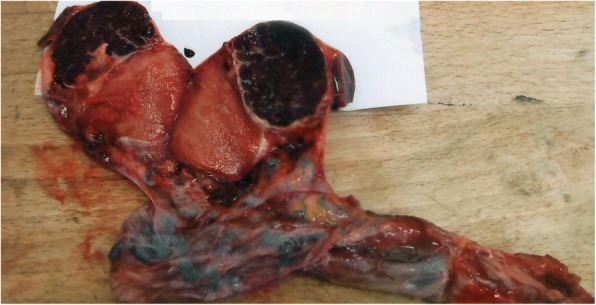
The macroscopic aspect of the suspect mass looks similar to splenic tissue

He underwent a radical inguinal orchiectomy. We first performed a high ligation of the spermatic cord. The operative specimen included the testis and the tunica vaginalis in one piece (Fig. 2). The macroscopic aspect of the supratesticular mass looks similar to splenic tissue (Fig. 3). There were no macroscopic lesions of the testis and the spermatic cord. His postoperative course was uneventful. He was discharged on the second postoperative day.

**Fig. 4:**
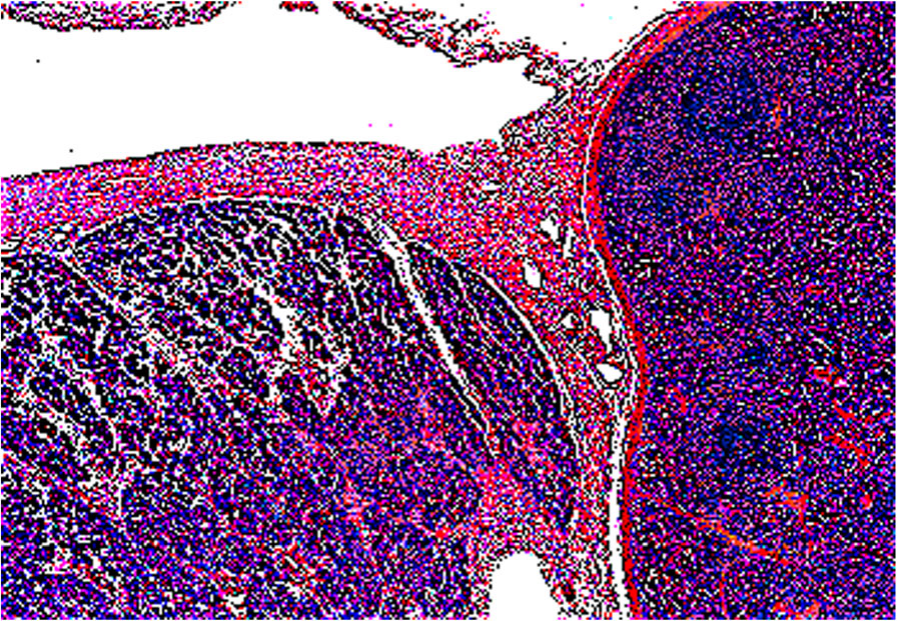
Histology of the operative specimen: regular splenic proliferation, independent from the testis and its adnexa. Splenic tissue is on the *right*, limited with a regular capsule. Testicular parenchyma is on the right, with an intact albuginea

Histological examination of the operative specimen confirmed the presence of regular splenic tissue in the suspect mass, without any signs of malignancy. The splenic proliferation had its proper and regular capsule, demarcating it from the testis. Testicular pulp, the albuginea and the tunica vaginalis had a preserved microscopic architecture (Fig. 4). He was examined 3 weeks after orchiectomy and he was examined again 2 months after the orchiectomy in our out-patient unit. An abdominal ultrasound showed an habitual location with standard measurements and a regular aspect of his spleen.

## Discussion

Splenogonadal fusion is a rare congenital anomaly, first described in 1883 by Boestrom [1]. Since then, around a 150 cases have been reported. It is mainly observed in adolescents and young adults. Association with other congenital malformations is possible, mainly cryptorchidism, limb defects, and micrognathia [2].

It occurs generally on the left side. Splenogonadal fusion is not exclusively observed in male patients. A few cases were reported in female patients. It is underdiagnosed in female patients, as ovaries are less accessible to physical examination [3].

Two types, with an equal frequency, were described by Putschar and Manion [4]: the continuous and the discontinuous form. In the continuous type, a cord of splenic or fibrotic tissue links the spleen to the ectopic gonad. In the discontinuous type, there is no connection to the spleen. An ectopic splenic tissue is annexed to the gonad, inside the tunica vaginalis, with a distinct capsule [5, 6].

The clinical presentation is not specific and diagnosis is often made on histological examination of the operative specimen. The discontinuous form usually presents as a hard scrotal nodule, mimicking a testicular tumor. In some cases, scrotal swelling is associated with specific splenic conditions, such as infectious mononucleosis or salmonellosis [7]. A bowel obstruction may reveal the discontinuous form. Operative findings in groin exploration or abdominal laparoscopy for cryptorchidism may also aid diagnosis [8].

Scrotal ultrasonography is not accurate enough preoperatively; as sonographic aspects are various, sensitivity and specificity are not high [9]. Magnetic resonance imaging is associated with the same shortcomings. In the continuous form, the linking cord between the spleen and the ectopic testis may be visualized [10]. Splenic scintigraphy using technetium-99m (^99m^Tc) is a valuable option once splenogonadal fusion is suspected. Radioactive tracer fixation in the spleen and the suspect mass is similar, confirming the ectopic splenic origin of the testicular appended mass [2].

Even if splenogonadal fusion is almost always a benign condition, surgery is still mandatory. In fact, histological examination of the operative specimen ensures formal diagnosis and rules out the infrequent association with testicular malignancies [11]. The association with a testicular neoplasm was described in only four cases [4]. There is no evident causality between splenogonadal fusion and malignant transformation. The rare observed cases were probably prone to develop a testicular neoplasm as they also presented cryptorchidism [12].

Even if imaging affords a better prediction and diagnostic orientation, many patients still undergo unnecessary orchiectomies. Once operative aspects are known, a surgeon can opt for conservative treatment, saving the testis [13]. In the case we report, our patient underwent an avoidable radical orchiectomy. An extemporaneous histological examination could have been useful to adjust the surgical attitude and preserve the testis.

## Conclusions

A better knowledge of splenogonadal fusion may increase the preoperatively indicated and diagnosed cases, in order to reassure patients and adjust the therapeutic attitude. Surgery remains essential, as malignancy must be ruled out. More specific imaging features and extemporaneous histological examination enable avoidance of an unnecessary orchiectomy.
